# Intestinal Injury After Suprapubic Catheterisation: A Scoping Review

**DOI:** 10.7759/cureus.74057

**Published:** 2024-11-19

**Authors:** Farhan Jarral, Abdelrahman Hamdy, Osama Abusand, Rosa Mobayen, Evripidis Tokidis

**Affiliations:** 1 Urology, Doncaster Royal Infirmary, Doncaster, GBR; 2 General Surgery, Doncaster Royal Infirmary, Doncaster, GBR; 3 Urology, Chesterfield Royal Hospital, Chesterfield, GBR; 4 Urology, Doncaster and Bassetlaw Teaching Hospitals NHS Trust, Doncaster, GBR; 5 Colorectal Surgery, Sheffield Teaching Hospitals, Sheffield, GBR

**Keywords:** bowel injury, complication management, literature summary, scoping review, suprapubic catheter

## Abstract

Suprapubic catheterisation (SPC) is a commonly performed urological procedure. Although it is generally safe, SPC-induced bowel injury is a rare but morbid complication. It is described in the literature, but management consensus is lacking. A scoping review was conducted assessing existing literature regarding the management of intestinal injury. The review highlighted that bowel perforation, particularly involving the small bowel and terminal ileum, is the commonest SPC-related bowel injury type. Depending on the severity of the injury and the patients' condition, various management strategies, ranging from exploratory laparotomy to less invasive techniques like laparoscopic intervention, are documented. Despite the introduction of preventive measures, such as ultrasound guidance, intestinal injury occurs. SPC-associated bowel injury is a serious but rare complication despite available preventative measures. Its management varies and depends on the part of the bowel injured and its severity. This review highlights reported management strategies specific to this injury and a literature summary to aid future quality improvement on the topic.

## Introduction and background

Suprapubic catheterisation (SPC) is a common emergency and long-term bladder drainage procedure [1]. The clinical practice of the technique of SPC insertion varies; however, after a large UK audit, the British Association of Urological Surgeons recommends ultrasound-guided SPC insertion or open cystostomy to reduce the risk of bowel injury [2]. Despite SPC’s widespread use, traumatic (or penetrating) bowel injury remains uncommon [3]. Nevertheless, its serious morbidity and potential mortality should be considered [4].

Clinical manifestations of SPC-related bowel injury can be subtle, specifically in comorbid patients for whom SPC is commonly indicated [2]. If bowel injury is suspected, urgent CT imaging should be performed, provided it is accessible in a timely manner [5]. Clinicians should also be mindful of the potential for late presentations, including during the initial catheter exchange [6]. UK guidelines advocate for providing patients and their carers with written instructions advising prompt re-referral and evaluation in the event of persistent or generalised abdominal pain, vomiting, or systemic symptoms [7]. Despite intestinal injury post-SPC insertion being a recognised complication, summarised evidence for the operative details of surgical management is lacking.

This scoping review aims to summarise the existing literature on SPC insertion-related bowel injury and the subsequent operative management strategies to aid future quality improvement work on the topic.

Materials and methods

This scoping review followed the PRISMA-ScR guidelines [8] and Arksey and O’Malley’s five-stage framework [9].

Research Questions

What parts of the bowel are injured, and to what extent?

What management strategies are currently used for SPC-associated bowel injury?

What are the outcomes of different SPC-related bowel injury management strategies?

On 01/07/2024, systematic searches were conducted in Embase and Medline via Ovid. Keywords included "suprapubic catheter," "complications," "management," "bowel injury," "infection," and relevant MeSH terms. The search was limited to studies published in English.

Inclusion Criteria

Published cases and studies focusing on surgical management of SPC-related bowel injury in adults and published studies in the English language were included.

Exclusion Criteria

Non-English language articles, editorials, reviews, and conference abstracts and studies where SPC was not the primary focus or where relevant data cannot be extracted were excluded.

Two independent reviewers (HA and FJ) screened titles and abstracts, followed by full-text reviews. Disagreements were resolved by consensus between the reviewers (HA and FJ) and when that was not possible, then by a third reviewer (ET).

Using a standardised Excel proforma, the data were charted by two independent reviewers (HA and FJ). Extracted information includes study title, author, year, country, design, sample size, SPC techniques, bowel injury classification as per The American Association for the Surgery of Trauma [10], management strategies, and key findings. The key findings were analysed to identify recurring themes regarding the management of SPC-induced bowel injury.

## Review

Results

**Figure 1:**
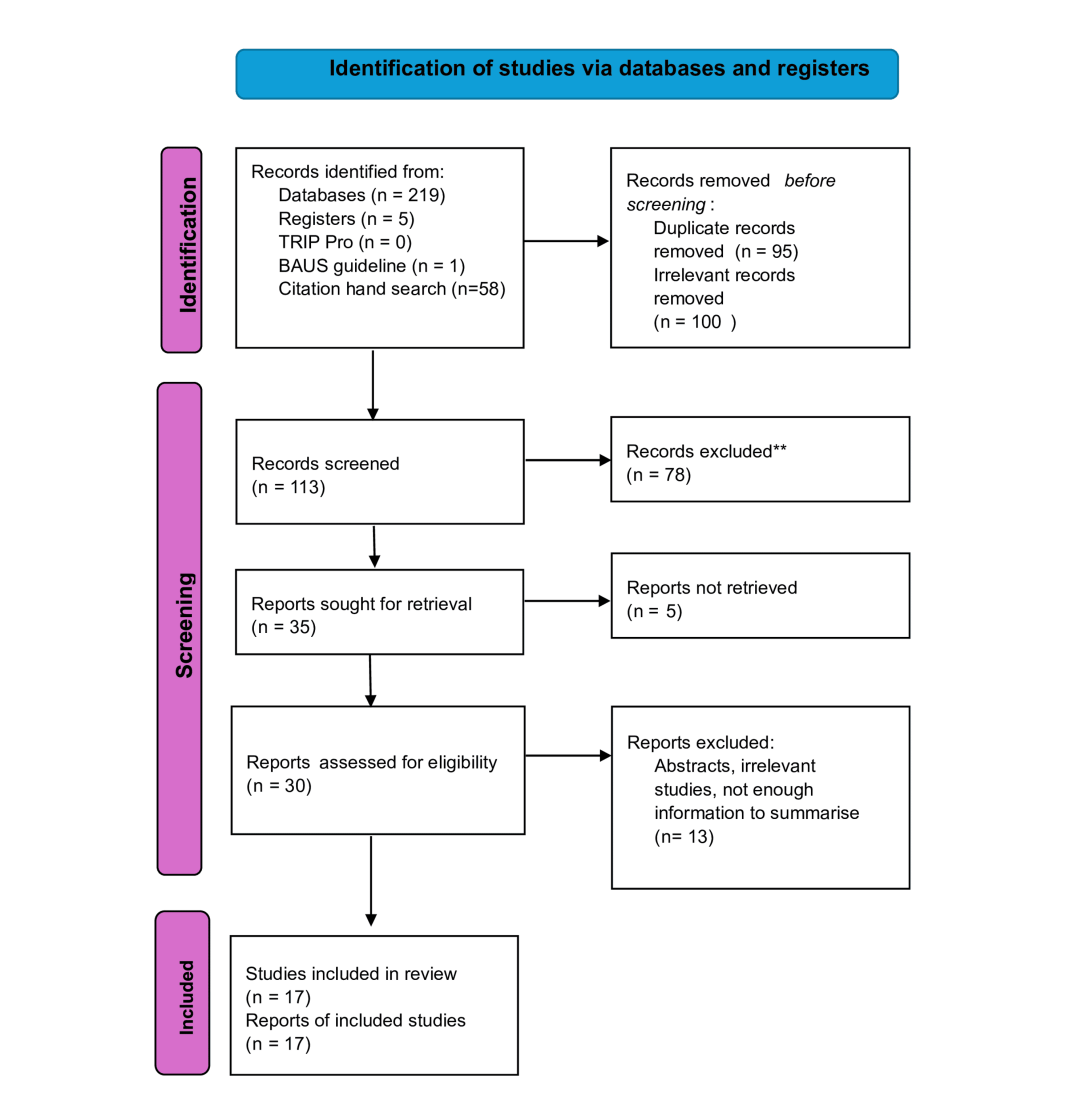
PRISMA flow diagram TRIP PRO: Turning Research Into Practice Professional; BAUS: British Association of Urological Surgeons; PRISMA: Preferred Reporting Items for Systematic Reviews and Meta-Analyses

The review included 17 studies (Figure 1). These comprise case reports and series exploring SPC-associated bowel injury. The studies, which date from 1995 to 2023, originate from multiple countries. Table 1 provides an overview of the study characteristics, caseload, SPC placement technique, type and Grade of bowel injury [10], and management approach.

**Table 1:** Summary of included studies SPC: Suprapubic catheterisation

Authors	Study date	Country	Study type	Case load	Surgical technique	Bowel injury type	Management
Foran et al. [6]	2018	Ireland	Case series	2	1) Flexible cystoscopy assisted and 2) Open replacement	1) Ileal perforation (Grade 2) and 2) Ileal perforation (Grade 2)	Laparotomy and small bowel resection
Chitale and Irving [11]	2010	UK	Case report	1	Unguided insertion	Sigmoid perforation (Grade 3)	Segmental resection
Wang et al. [12]	2021	China	Case report	1	Open replacement of SPC	Small bowel perforation (Grade 3)	Conservative management
Verma et al. [13]	2020	India	Case report	1	Unguided insertion	Distal ileum sealed perforation (Grade 3)	Laparotomy and small bowel resection and ileostomy
Chang et al. [14]	2012	Taiwan	Case report	1	Open replacement	Terminal ileal perforation (Grade 3)	Laparotomy and small bowel resection and ileostomy
Jackson et al. [15]	2010	UK	Case report	1	Unguided insertion	Small bowel injury (Grade 3)	Conservative management
Stonier et al. [16]	2017	UK	Case report	1	Ultrasound-guided and flexible cystoscopy-assisted	Terminal ileum perforation (Grade 3)	Laparotomy and small bowel resection
Murugesan and Madhavan [17]	2023	India	Case report	2	Ultrasound-guided suprapubic catheter insertion	Ileal perforation (Grade 3)	1) Conservative and 2) Laparotomy and primary repair
Rajmohan et al. [18]	2013	Germany	Case report	1	Unguided insertion	Rectal perforation (Grade 3)	Laparoscopic primary repair
Gallagher et al. [19]	2013	UK	Case report	1	Cystoscopy-guided replacement	Small bowel injury (Grade 2)	Conservative management
Kass-Iliyya et al. [20]	2012	UK	Case report	1	Unguided replacement	Descending colon injury (Grade 3)	Conservative management
Gajera and Hong-Chae [21]	2011	USA	Case report	1	Under direct visualisation (open)	Sigmoid colon injury (Grade 3)	Diverting loop colostomy
Wu et al. [22]	2007	Taiwan	Case report	1	Not mentioned	Terminal ileal perforation (Grade 3)	Laparotomy and small bowel resection
Ananthakrishnan et al. [23]	2006	UK	Case report	1	Cystoscopy-guided insertion	Mesenteric injury (Grade 2)	Laparotomy and replacement of SPC and closure of mesentery
Ahmed et al. [24]	2004	UK	Case report	1	Ultrasound-guided supra-pubic catheter insertion	Small bowel injury (Grade 3)	Laparotomy and small bowel resection
Witham et al. [25]	2002	UK	Case report	1	Cystoscopic-guided insertion	Colonic perforation (Grade 3)	Laparotomy and repair of colovesical fistula

The SPC placement techniques described include ultrasound-guided, open, unguided, and flexible cystoscopy-assisted insertion. Ultrasound guidance is the most frequently mentioned method. Small bowel injuries identified in the studies encompass primarily ileal perforations. Colonic perforations were documented in five cases. The injuries are classified using a grading system, with examples ranging from Grade 2 to 3.

Management strategies for bowel injuries vary according to the severity. Conservative management is applied in some instances, while surgical interventions, including laparotomy, bowel resection, and primary repair or anastomosis, are documented in more severe cases. Examples of surgical management include exploratory laparotomy and resection with primary anastomosis, reflecting diverse approaches depending on injury severity. The outcomes following treatment were documented in most of the cases. Sixteen cases resulted in full recovery after intervention, while three cases experienced complications such as infection or adhesion, necessitating further medical management. There was one case of mortality, which was attributed to delayed diagnosis and intervention of the bowel injury.

Discussion 

The scoping review provides a comprehensive overview of the current evidence on surgical management of SPC-associated bowel injury. It highlights known variations in SPC placement techniques and grades of bowel injury [3]. Surgical intervention emerged as the predominant method for managing these injuries, with a high rate of successful recovery observed when timely intervention was provided. Only one case resulted in death [11], underscoring the importance of prompt diagnosis and treatment.

The review confirms previous study findings that while SPC is generally considered a safe and effective procedure for bladder drainage, it is not without significant risks [3]. Bowel perforation is SPC’s most serious complication, with several case reports in the literature. These perforations most commonly involve the small bowel, particularly the terminal ileum, and can result in severe outcomes such as peritonitis and sepsis if not promptly recognised and managed.

The management of SPC-related complications often necessitates surgical intervention, particularly in cases of bowel perforation. Most of the cases in the scoping review utilised exploratory laparotomy with resection of the affected bowel segments. In one case, less invasive techniques such as laparoscopic management have been employed [18], although the choice of intervention largely depended on the severity of the injury and the patient’s overall condition.

The review also highlights the importance of early detection and prompt management of SPC complications. Clinical suspicion is essential, particularly in the immediate postoperative period, where signs of poor catheter drainage, abdominal pain, and peritonitis may indicate a serious underlying complication [3]. The use of imaging modalities such as computed tomography is critical in confirming the diagnosis and guiding the appropriate surgical intervention [5]. The role of previous abdominal surgeries as a significant risk factor for bowel injury during SPC insertion. Patients with a history of laparotomies or other lower abdominal surgeries are at a higher risk due to the presence of adhesions, which can alter normal anatomy and increase the likelihood of the bowel being inadvertently punctured during catheter insertion [21]. Moreover, the review did not identify the presence of a general surgeon during the operative management of SPC-induced bowel injury. Future quality improvement work should focus on developing a multi-speciality consensus for managing SPC-induced bowel injury.

Limitations

This review includes case reports and series only that described the operative interventions. As this is a scoping review, providing insights into the details of the surgical management, the case series by Hall et al. [3], Sheriff et al. [26] and Ahluwalia et al. [27] were not included as they only quote bowel injury numbers rather than specific details on the management. The case series by Cronin et al. [28] describe bowel injury in two cases but did not include enough details for the purpose of this review. 

## Conclusions

This scoping review synthesised the available literature on the surgical management of SPC-induced bowel injury, highlighting areas for future quality improvement work. In conclusion, while SPC remains a valuable tool in the management of bladder drainage, bowel injury, despite its low incidence, is a serious complication, and its surgical management requires close attention. The review highlights the importance of adherence to preventive strategies, vigilance for early detection, and prompt multidisciplinary management.
